# Evolutionary History and Genome Organization of DUF1220 Protein Domains

**DOI:** 10.1534/g3.112.003061

**Published:** 2012-09-01

**Authors:** Majesta S. O’Bleness, C. Michael Dickens, Laura J. Dumas, Hildegard Kehrer-Sawatzki, Gerald J. Wyckoff, James M. Sikela

**Affiliations:** * Department of Biochemistry and Molecular Genetics, Human Medical Genetics and Neuroscience Programs, University of Colorado School of Medicine, Aurora, Colorado 80045; † Institute of Human Genetics, University of Ulm, 89081 Ulm, Germany, and; ‡ Division of Molecular Biology and Biochemistry, School of Biological Sciences, University of Missouri, Kansas City, Missouri 64110

**Keywords:** DUF1220, *NBPF*, *PDE4DIP*

## Abstract

DUF1220 protein domains exhibit the most extreme human lineage–specific (HLS) copy number increase of any protein coding region in the human genome and have recently been linked to evolutionary and pathological changes in brain size (*e.g.*, 1q21‐associated microcephaly). These findings lend support to the view that DUF1220 domain dosage is a key factor in the determination of primate (and human) brain size. Here we analyze 41 animal genomes and present the most complete account to date of the evolutionary history and genome organization of DUF1220 domains and the gene family that encodes them (*NBPF*). Included among the novel features identified by this analysis is a DUF1220 domain precursor in nonmammalian vertebrates, a unique predicted promoter common to all mammalian *NBPF* genes, six distinct clades into which DUF1220 sequences can be subdivided, and a previously unknown member of the *NBPF* gene family (*NBPF*25). Most importantly, we show that the exceptional HLS increase in DUF1220 copy number (from 102 in our last common ancestor with chimp to 272 in human; an average HLS increase of ∼28 copies every million years since the *Homo/Pan* split) was driven by intragenic domain hyperamplification. This increase primarily involved a 4.7 kb, tandemly repeated three DUF1220 domain unit we have named the *HLS DUF1220 triplet*, a motif that is a likely candidate to underlie key properties unique to the *Homo sapiens* brain. Interestingly, all copies of the *HLS DUF1220 triplet* lie within a human-specific pericentric inversion that also includes the 1q12 C‐band, a polymorphic heterochromatin expansion that is unique to the human genome. Both cytogenetic features likely played key roles in the rapid *HLS DUF1220 triplet* hyperamplification, which is among the most striking genomic changes specific to the human lineage.

Genome sequences encoding DUF1220 protein domains have undergone an exceptional human lineage-specific (HLS) increase in copy number that decreases generally as a function of a species\x{2019} evolutionary distance from humans (Popesco *et al*. 2006). The HLS DUF1220 copy number expansion was first identified as part of genome-wide survey of gene copy number change among human and great ape lineages (Fortna *et al*. 2004). An independent study identified the same gene family and named it neuroblastoma breakpoint family (NBPF) when a member of the family was found to be disrupted by a rearrangement in a neuroblastoma patient (Vandepoele *et al*. 2005). DUF1220 domains are approximately 65 amino acids in length and are encoded by sequences that show signs of positive selection and, in brain, exhibit neuron-specific expression (Popesco *et al*. 2006; Dumas *et al*. 2007). Notably, DUF1220 sequences are found in two dissimilar genomic environments: as a single, likely ancestral, domain encoded by the single- or low-copy PDE4DIP (myomegalin) gene, and separately, as tandemly repeated copies encoded by members of the NBPF gene family. It is only this latter form that underwent the dramatic copy number amplification in recent primate evolution (Vandepoele *et al*. 2005; Popesco *et al*. 2006).

DUF1220 protein domains have also generated interest because copy number variations (CNVs) in the 1q21.1 region, where most DUF1220 sequences map, have been implicated in numerous recurrent human developmental and neurogenetic diseases (Dumas and Sikela 2009). These include microcephaly and macrocephaly (Brunetti-Pierri *et al*. 2008; Velinov and Dolzhanskaya 2010), autism (Autism Genome Project Consortium 2007; Pinto *et al*. 2010), schizophrenia (International Schizophrenia Consortium 2008; Levinson et al. 2011), mental retardation (Mefford *et al*. 2008; Jaillard *et al*. 2010), congenital heart disease (Christiansen *et al*. 2004; Greenway *et al*. 2009), congenital anomalies of the kidney and urinary tract (Weber *et al*. 2011), and neuroblastoma (Vandepoele *et al*. 2008; Diskin *et al*. 2009). Interestingly, a recent study has implicated DUF1220 domain copy number loss in the etiology of 1q21-associated microcephaly, and provides support for the view that DUF1220 copy number may function as a key general effecter of evolutionary, pathological, and normal variation in brain size among primate (and human) lineages (Dumas *et al*. 2012).

These findings are also consistent with a model which proposes that 1) the strong selection pressures that drove the rapid HLS increase in DUF1220 copies favored retention of the high genomic instability of the 1q21 region, and 2) it is the resulting unstable, duplication-rich genomic architecture that is a major factor in the etiology of the numerous 1q21.1 disorders that have been reported (Dumas and Sikela 2009). Given these observations, we undertook a comprehensive reconstruction of DUF1220 evolutionary history to gain insight into both the key genomic events that underlie the rapid evolutionary expansion in DUF1220 copy number as well as how this process may be responsible for the multitude of recurrent genetic and developmental diseases associated with the 1q21.1 region.

## Methods

### Genome searches and nucleotide alignments

DUF1220 nucleotide and protein domains, NBPF proteins, and PDE4DIP proteins were used as BLAT/BLAST queries against 40 genomes in the UCSC (Kent *et al.* 2002), Ensembl (Flicek *et al.* 2010), or NCBI (Pruitt *et al.* 2009) genome databases, and proteins were searched against the NCBI protein database using HMMER3 (Finn *et al.* 2011). BLAT results were considered significant with an alignment of at least 50 and a score of at least 100 and BLAST results with an alignment of 50 and a score of at least e^−10^. All significant NBPF alignment results were additionally investigated using the Pfam (Finn *et al.* 2010) protein database to determine homology to DUF1220 domains at the protein level. Homology was considered significant with a score of e^−5^.

DUF1220 domain precursor regions were found by aligning the human *PDE4DIP* DUF1220 seed domain to the predicted PDE4DIP proteins from five genomes, including zebrafish, *Xenopus tropicalis* (frog), *Anolis carolinensis* (lizard), chicken, and opossum, from the UCSC genome browser using ClustalW (Larkin *et al.* 2007). The aligned region was then extracted from the protein, the DUF1220 precursors were realigned, and a phylogeny was created using the ClustalW program. Confirmation of the DUF1220 precursor region was performed by HMMER3 (Finn *et al.* 2011).

Two thousand base pairs upstream of the human *NBPF4* gene was used as a BLAT query against human as well as other mammalian genomes in the UCSC and Ensembl genome browsers to determine whether a conserved *NBPF* promoter sequence was present within and across multiple genomes. The two thousand base pairs upstream of predicted *NBPFs* in eight mammalian genomes were extracted and then aligned using the VISTA global alignment tool (Frazer *et al.* 2004). These same regions were then searched for conserved transcription factor binding sites from the TRANSFAC database using rVISTA 2.0 (Loots and Ovcharenko 2004).

### Evolutionary analyses

The DUF1220 phylogenetic profile was inferred using the minimum evolution (ME) method (Rzhetsky and Nei 1992). The consensus tree inferred from five optimal trees is shown (Figure 2A). Branches corresponding to partitions reproduced in less than 50% trees are collapsed. The evolutionary distances were computed using the Poisson correction method (Zuckerkandl and Pauling 1965) and are in the units of the number of amino acid substitutions per site. The ME tree was searched using the close-neighbor-interchange (CNI) algorithm (Nei and Kumar 2000) at a search level of 1. The neighbor-joining algorithm (Saitou and Nei 1987) was used to generate the initial tree. All positions containing alignment gaps and missing data were eliminated only in pairwise sequence comparisons (pairwise deletion option). There were a total of 57 positions in the final dataset. Phylogenetic analyses were conducted in MEGA4 (Tamura *et al.* 2007).

The HLS triplets (HLS1-HLS2-HLS3) for each *NBPF* gene in the 1q21 were aligned using PRANK (Löytynoja and Goldman 2005). The HLS triplets were defined as the genomic sequence starting from the small exon of an HLS1 DUF1220 within an *NBPF* gene, across the following HLS2 and HLS3 sequences, and to the beginning of the next downstream HLS1 small exon. The phylogenetic tree of these alignments was generated using the APE (Paradis *et al.* 2004) package in R.

### *NBPF* annotation, copy number organization, and ontogeny

Human *NBPF* genomic positions were determined by a combination of promoter start positions, the UCSC and Ref-Seq gene tracks at the Santa Cruz genome browser, corresponding DUF1220 domain positions, and the Ensembl gene prediction engine (specifically for *NBPF* genes predicted by promoter and DUF1220 domain position, but not predicted by the UCSC or Ref-Seq gene tracks). Chimp *NBPF* numbers were predicted using a combination of the number of predicted *NBPF* start positions when the promoter sequences were utilized as BLAT queries, the number of DUF1220 sequences sharing clades with known human and orangutan DUF1220 domains in the phylogenetic analysis, and parsimony based upon shared *NBPF* genes between orangutan and human genomic assemblies. The number of DUF1220 domains in the common ancestor of *Pan* and human was determined by subtracting the number of DUF1220 domains in chimp-specific clade expansions from the chimp genome DUF1220 count.

The organization and evolution of each *NBPF* gene in the human genome was evaluated based upon the phylogeny generated. A superclade was considered conserved if 50% or more of the clade was made up of nonhuman DUF1220 domains and it contained DUF1220 domains from all five primates. A superclade was classified as HLS if more than 60% of the clade was made up of human DUF1220. The superclade classification used is further supported by the fact that DUF1220 members of each clade hold a particular placement within the organization of an *NBPF* gene (supporting information, Table S1) and each clade is representative of one or more Pfam DUF1220 seed domains (Table S2). Determination of whether a human-specific DUF1220 domain arose either via gene duplication or domain amplification was determined by clade analysis. A DUF1220 domain that clusters with a domain from another gene was determined to have arisen via gene duplication. A DUF1220 domain that clusters with domains within the same gene was determined to have arisen via domain amplification. Timing of appearance of CON1 and CON2 domains is based on amino acid alignments of nonprimate mammalian DUF1220 domains with the CON1 and CON2 clade domains (data not shown). *PDE4DIP*, *NBPF*, and DUF1220 domain chromosomal positions in human, orangutan, macaque, marmoset, cow, dog, horse, and pig were determined using the UCSC genome browser. Chimp chromosome 1 positions were determined using the UCSC genome browser as well as inversion data mapped by Szamalek *et al.* (2006).

The DUF1220 domain evolutionary chronology was assembled based on timing of events found by the analysis of 41 animal genomes, the CM promoter analysis performed here, and timing of DUF1220 clades based on the phylogenetic profile. The chronology also incorporates the work from Vandepoele *et al.* (2009) on the acquisition of the EVI5 promoter and the altered expression work from Illarionova *et al.* (2007) on the incorporation of an intronic HERV(K) insertion.

## Results

### DUF1220 origin and copy number expansion

**Figure 1:**
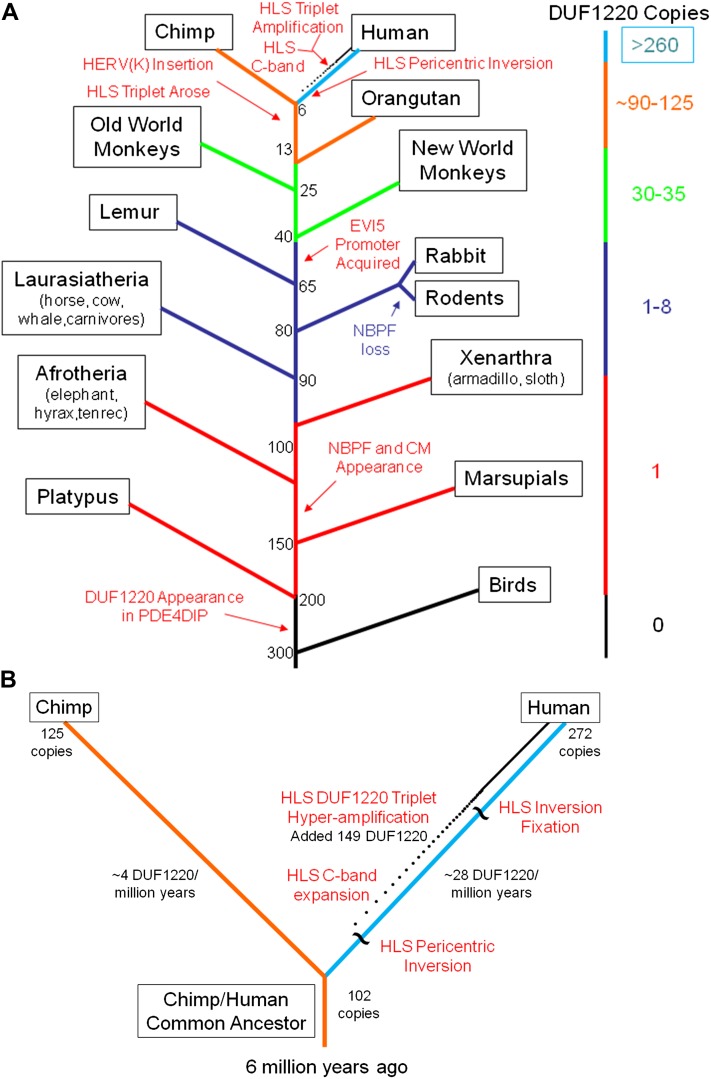
(A) Phylogenetic tree of the major events shaping the evolution of the DUF1220 domain family. Depicted are different mammalian lineages with their approximate divergence times at branch points in millions of years ago. Major events in DUF1220 evolution are identified, and approximate numbers of DUF1220 domains per genome are shown on the right. The dotted line of increasing density along the human divergence branch is indicative of the accumulation of the *HLS DUF1220 triplet* since the HLS pericentric inversion. (B) Expanded image of the human/chimp split in the evolutionary timeline of DUF1220 domains showing the different evolutionary fates of DUF1220 domains within each of the two lineages.

Using genome sequences of 41 animal species (36 mammals and 5 nonmammalian vertebrates), we have generated the most complete evolutionary history of DUF1220 domains from their first appearance to their current levels in existing lineages (Table 1). These analyses indicate that the DUF1220 protein domain first appears as part of the *PDE4DIP* gene at least 200 million years ago (mya) (Figure 1). Although almost all vertebrates sequenced to date have homologs of PDE4DIP, the proteins do not show levels of sequence conservation high enough to be formally classified by BLAT/BLAST as containing a DUF1220 domain until the emergence of the mammalian lineages. However, the homologous protein coding region of the PDE4DIP DUF1220 domain in nonmammalian vertebrates shows 70% similarity and 32% identity at the amino acid level as far back as bony fish (Figure S1), establishing that a DUF1220 domain precursor in PDE4DIP existed at least 450 mya. This was confirmed by an HMMER (Finn *et al.* 2011) search against the NCBI protein database. These findings indicate that the DUF1220 protein domain precursor likely existed as an important functional entity prior to the emergence of the mammalian order and persisted as the only DUF1220 domain form for 50–100 million years.

**Table 1:** Genomes searched for DUF1220 domains, NBPF genes, and *PDE4DIP*

Genome	Coverage	PDE4DIP	Total DUF1220	NBPF Genes	Source	Assembly	Promoter
Euarchotanglires							
Human	>9X	2	272	23	UCSC	GRCh37	CM,EVI5, HERV
Chimp	4X + WGS	3	125	19	UCSC + Ensembl	panTro2	CM,EVI5, HERV
Gorilla	2X + Illumina	3	99	15	Ensemble	gorGor3	CM,EVI5, HERV
Orangutan	6X	4	92	11	UCSC + Ensembl	ponAbe2	CM,EVI5
Gibbon	5.6X	3	53	10	Ensembl	nomLeu1	CM,EVI5
Macaque	High-BAC	1	35	10	UCSC + Ensembl	rheMac2	CM,EVI5
Marmoset	6X + WGS	1	31	11	UCSC	calJac3	CM,EVI5
Mouse lemur	1.93X	1	2	1	Ensembl	micMur1	Seq gap
Bushbaby	1.5X	1	3	2	Ensembl	otoGar1	
Tarsier	1.83X	1	1	0	Ensembl	tarSyr1	
Rabbit	7X	1	8	3	UCSC & Ensembl	oriCun2	CM
Pika	1.93X	1	1	0	Ensembl	ochPri1	
Mouse	High-BAC	1	1	0	UCSC	mm9	
Rat	High-WGS	1	1	0	UCSC	rn4	
Guinea pig	6.79X	1	1	1	UCSC & Ensembl	cavPor3	
Squirrel	1.9X	1	1	1	Ensembl	speTri1	
Tree shrew	2X	1	4	3	Ensembl	tupBel1	CM
Laurasiatheria							
Cow	7X	1	7	3	UCSC & NCBI	bosTau6	CM
Dolphin	2.59X	1	4	1	Ensembl	turTru1	CM
Pig	4X	1	3	1	UCSC & Ensembl	susScr2	CM
Horse	6.79X	1	8	3	UCSC & NCBI	equCab2	CM
Dog	2005 WGS	1	3	1	UCSC	canFam2	CM
Panda	56X Illumina	1	2	1	UCSC	ailMel1	CM
Cat	2.8X	1	3	2	UCSC & Ensembl	felCat4	CM
Megabat	2.63X	1	1	0	Ensembl	pteVam1	
Microbat	1.7X	1	1	0	Ensembl	myoLuc1	
Hedgehog	1.86X	1	1	0	Ensembl	eriEur1	
Shrew	1.9X	1	1	0	Ensembl	sorAra1	
Xenarthra							
Armadillo	2X	1	1	0	Ensembl	dasNov1	
Sloth	2.05X	1	1	0	Ensembl	choHof1	
Afrotheria							
Elephant	7X	1	1	2	UCSC & Ensembl	loxAfr3	CM
Hyrax	2.19X	1	1	0	Ensembl	proCap1	
Tenrec	2X	1	1	0	Ensembl	echTel1	
Metatheria							
Opossum	7.33X	1	1	0	Ensembl	monDom5	
Wallaby	2X	1	1	0	Ensembl	Meug1	
Prototheria							
Platypus	6X	1	1	0	UCSC	ornAna1	
Other vertebrate							
Chicken	6.6X	0	0	0	UCSC	galGal3	
Zebra finch	6X	0	0	0	UCSC	taeGut1	
Lizard	6.8X	0	0	0	UCSC	anoCar1	
Frog	7.65X	0	0	0	UCSC	xenTro2	
Zebrafish	7X	0	0	0	UCSC	danRer6	

Of the 41 genomes searched, 36 were from mammals, and 5 were from nonmammalian vertebrates.

Investigation of 36 mammalian genomes [one Prototheria (platypus), two Metatheria (marsupials), and 33 Eutheria (placental mammals)] reveals that the first DUF1220 domain outside of PDE4DIP appeared ∼100–150 mya when the domain likely underwent a duplicative transposition into a new genomic environment. This event generated a new DUF1220 domain copy separate from the *PDE4DIP* gene and created a new gene family (*NBPF*) that exclusively encodes DUF1220 protein domains. This timeframe is based on homologs of *NBPF* genes found in three of the four sister superorders of placental mammals: Afrotheria (elephant), Laurasiatheria (hoofed mammals and carnivores), and Euarchotanglires (supraprimates). A complete list of the DUF1220 domains found among all 36 mammals investigated is given in Table 1.

It should be noted that the Rodentia order has no *NBPF* gene homologs in the mouse, rat, Guinea pig, or squirrel genomes and only carries the single DUF1220 domain that is found in the *PDE4DIP* gene. The most parsimonious explanation for this absence is that *NBPF* homologs were lost in a common ancestor of the rodents. This conclusion is based on two observations: (1) that there is a significant number of species that contain *NBPF* homologs that diverged prior to the emergence of the supraprimate superorder, and (2) that their sister order Lagomorpha (rabbit) has *NBPF* homologs.

In nonprimate mammals, copy number expansion of the NBPF type of DUF1220 domain was minimal (1–7 NBPF DUF1220 domains per genome), with the majority of DUF1220 domains located within 0.5 Mb of the *PDE4DIP* gene and thus consistent with a local duplication event. This is in sharp contrast to the dramatic changes that occurred in the primate order, which show a pattern of increasing DUF1220 copy number as a function of increasing phyletic proximity to human. This progressive expansion can be seen from lemur (2–3 copies) to new and old world monkeys (30–35 copies) to apes (90–125 copies) and finally to human (272 copies in the hg19 build) (Figure 1 and Table 1). The primate *NBPF* genes also show elevated DUF1220 copy number per gene (2–52 DUF1220 domains) as compared with the nonprimate mammalian *NBPF*s (1–4 DUF1220 domains), reflecting an enhanced level of intragenic domain amplification.

### Organization of DUF1220-encoding genes

Analysis of the HLS increase in DUF1220 domains is challenging due to the lack of a definitive *NBPF* gene annotation. Current DUF1220/*NBPF* annotations often lack defined gene start and stop points, and they frequently list individual *NBPF* genes at multiple locations on chromosome 1. Therefore, a detailed comparison of *NBPF* genes within and between species was undertaken to more precisely define *NBPF* genes and improve current annotation of the gene family.

#### Identification of the CM promoter:

Comparison of *NBPF* genes across eight species, including 2000 bp upstream of their predicted start positions, indicated that the promoter regions of human *NBPF*s 4–7 showed remarkable similarity to the promoter regions of many of the *NBPF*s in both primates and nonprimates (Figure S2). Alignment of these regions revealed that the 900 bp upstream of *NBPF* genes was particularly conserved, with the most divergent promoter regions (human and cow) having greater than 50% identity. When this region is searched *vs.* a transcription factor binding site database (TRANSFAC), two conserved binding motifs were detected: an OCT4 binding site, implicated in control of pleuripotent stem cell embryogenesis (Wang and Dai 2010), and an IRF1 binding site, implicated in induction of interferon, tumor suppression, cell-cycle control, and apoptosis (Romeo *et al.* 2002). We have designated this 900 bp conserved region as the CM promoter due to its conservation among all mammalian *NBPF* genes.

#### Specific criteria for defining NBPF genes:

When the CM promoter is used as a BLAT/BLAST query against the 41 mammalian genomes investigated, only three regions appear: (1) upstream regions of nonprimate *NBPF* genes, (2) upstream regions of the majority of primate *NBPF* genes, and (3) in the fourth intron upstream of the first DUF1220 domain in *NBPF* genes, where it is not in the promoter position. In the primate *NBPF* genes with an intronic CM promoter, the genes have acquired the previously documented *EVI5* promoter (Vandepoele *et al.* 2009). Interestingly, this shows that the CM promoter region is distinct from the *EVI5* promoter and may act as an alternative start site in primate *NBPF* genes that have acquired the *EVI5* promoter. This analysis indicates that all *NBPF* genes have either only the predicted CM promoter or both the EVI5 and CM promoters. Based on these data, we propose that the presence of the CM promoter immediately upstream of one or more predicted DUF1220 domains is criterion that represents the *sine qua non* for defining *NBPF* genes. This new definition allowed us to generate the most comprehensive annotation so far reported of the 23 *NBPF* genes in the human genome (Table 2). This list includes all previously documented *NBPF* genes as well as an undocumented gene predicted to encode 16 DUF1220 domains, which we have named *NBPF25*.

**Table 2:** Predicted *NBPF* genes in the human genome

Name	Position	No. of DUF1220	No. of DUF1220 in Triplet	Promoter	NBPF Type
NBPF1	16,890,412–16,939,982	7	0	EVI5/CM	Primate
NBPF2p	21,749,601–21,754,300	3	0	None	Pseudogene
NBPF3	21,766,631–21,811,392	5	0	EVI5/CM	Primate
NBPF4	108,765,963–108,786,703	4	0	CM	Nonprimate
NBPF5p	108,918,460–108,953,432	2	0	CM	Pseudogene
NBPF6	108,992,904–109,013259	4	0	CM	Nonprimate
NBPF7	120,377,389–120,387,779	2	0	CM	Nonprimate
**NBPF8**	**144,146,807**–**144,224,481**	**44**	**42**	**Gap**	**HLS**
NBPF9	144,593,696–144,828,810	7	0	EVI5/CM	Primate
**NBPF10**	**145,293,371**–**145,368,682**	**40**	**36**	**CM**	**HLS**
NBPF11	146,032,543–146,068,252	7	0	EVI5/CM	Primate
**NBPF25**	**146,214,651**–**146,253,110**	**16**	**11**	**Gap**	**HLS**
**NBPF12**	**146,374,056**–**146,467,744**	**34**	**29**	**EVI5/CM**	**HLS**
NBPF13	146,581,444–146,596,147	5	0	CM	Primate
NBPF24	147,574,324–147,624,601	7	0	EVI5/CM	Primate
**NBPF14**	**148,003,643**–**148,026,039**	**10**	**7**	**Gap**	**HLS**
**NBPF20**	**148,251,113**–**148,346,929**	**52**	**48**	**CM (? gap EVI5)**	**HLS**
NBPF15	148,556,090–148,596,267	6	0	EVI5/CM	Primate
NBPF16	148,739,442–148,758,311	6	0	CM (? gap EVI5)	Primate
NBPF23	149,089,947–149,154,938	6	0	EVI5/CM	Primate
NBPF18p	151,991,138–152,015,250	0	0	CM	Pseudogene
NBPF21p	Chr3: 36,657,498–36,678,949	1	0	None	Pseudogene
NBPF22p	Chr5: 85,578,262–85,593,362	2	0	CM	Pseudogene

This table was created using the new comprehensive annotation method described here. Listed are the *NBPF* name, genomic position, number of encoded DUF1220 domains, number of DUF1220 domains intragenically amplified in the HLS triplet clades, and predicted promoters. Genes containing amplified *HLS DUF1220 triplets* are in bold.

### DUF1220 phylogeny

**Figure 2:**
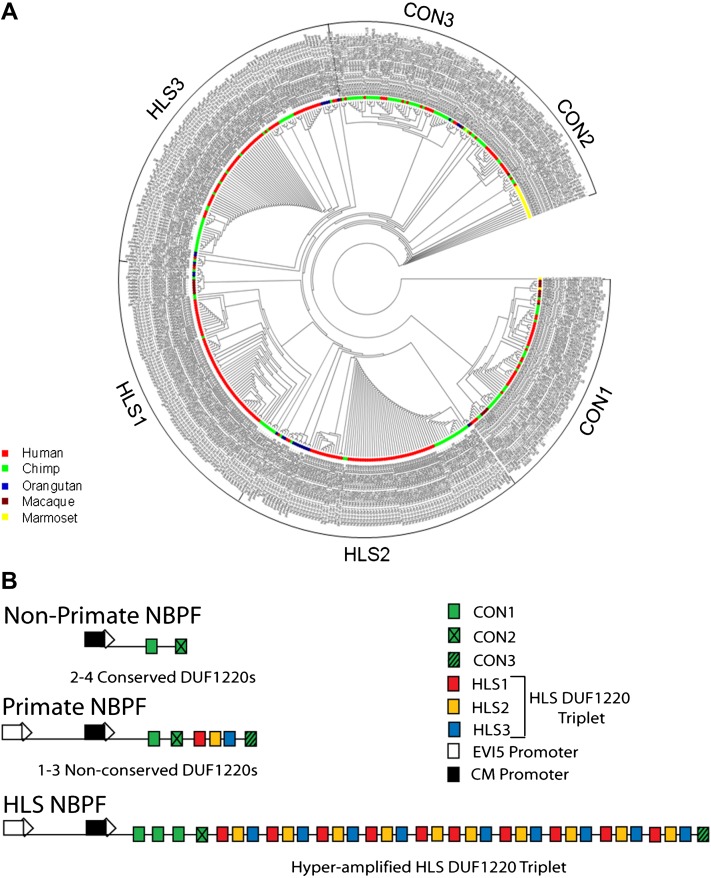
(A) Consensus tree of evolutionary relationships of 429 DUF1220 sequences. The evolutionary history was inferred using the Minimum Evolution method with the consensus tree inferred from five optimal trees. Species covered by the tree are color coded. The different types of DUF1220 clades are bracketed. (B) Gene organization of the three distinct types of *NBPF* genes. Top: Nonprimate mammal *NBPF* organization (NBPFs 4, 6, and 7). Middle: Typical primate *NBPF* organization (NBPFs 1, 3, 9, 11, 13, 24, 15, 16. and 23). Bottom: HLS *NBPF* organization (NBPFs 8, 10, 12, 25, 14, and 20).

**Figure 3:**
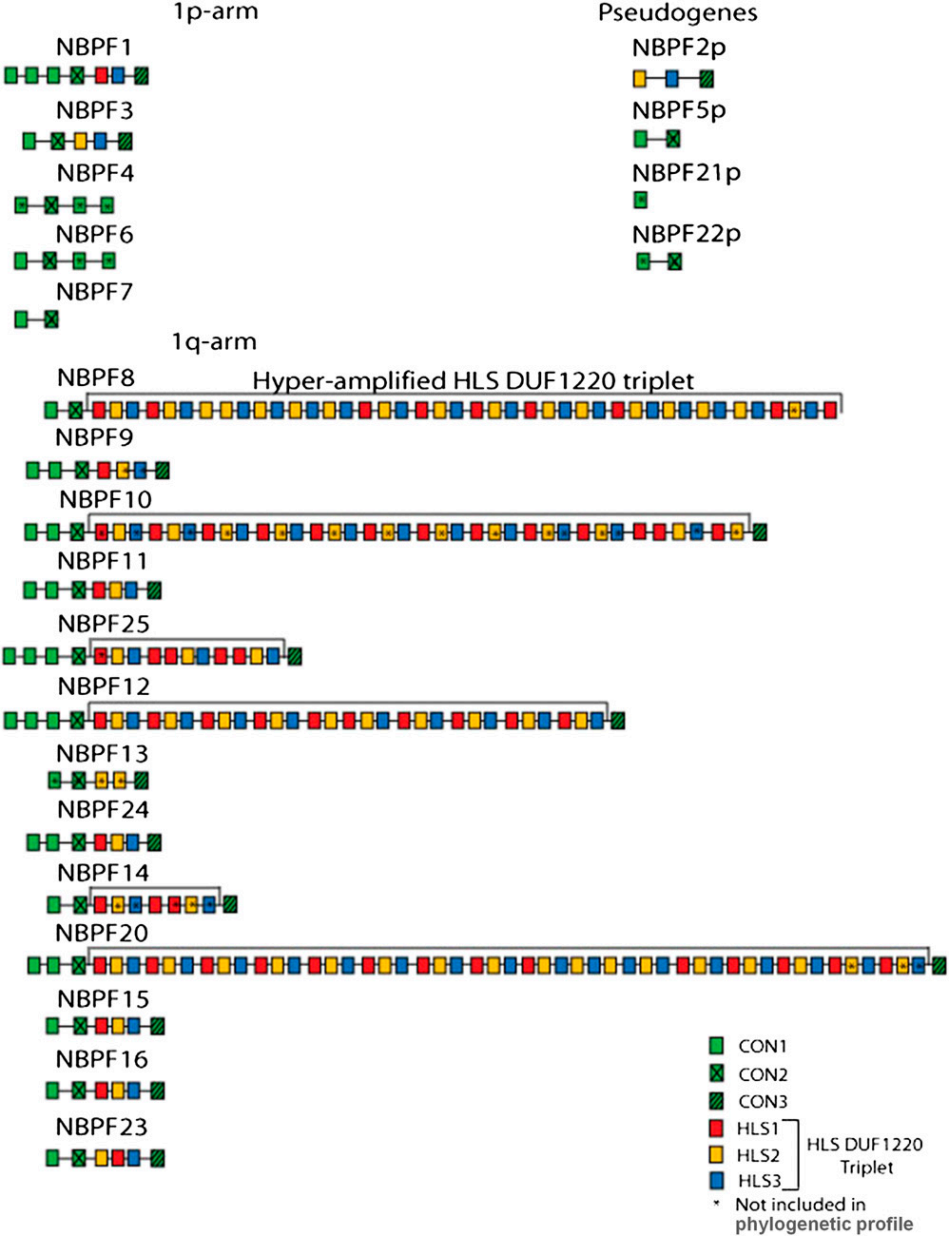
Gene organization of all *NBPF* genes in the human genome. The DUF1220 domains are shown as colored boxes that correspond to the different clades shown in Figure 2A. The hyperamplified *HLS DUF1220 triplets* are bracketed in the six human *NBPF* genes in which they occur.

To determine the evolutionary relationship of the DUF1220 domains within defined *NBPF* genes, a phylogenetic profile of DUF1220 evolution among primates was created for 429 predicted DUF1220 domains in the human, chimp, orangutan, rhesus macaque, and marmoset genomes (Figure 2A). This analysis indicates that DUF1220 sequences can be subdivided into six superclades, which can be distinguished by both their evolutionary conservation within the primate order as well as their position and order within each gene (Table S1). We have classified three of the six clades as conserved (CON1–3), as DUF1220 domains from all primates investigated have members of these three clades and greater than 50% of the DUF1220 copies in these clades are also found in species other than human. Interestingly, each CON clade maintains a distinct position within each *NBPF* gene: CON1 clade members occur at the N-terminus of all *NBPF* genes, CON2 members are adjacent to CON1 in all *NBPF* genes (this is the C-terminal DUF1220 domain in some NBPFs), and CON3 members reside at the C-terminus of the great majority of NBPF genes (Figures 2B and 3). Amino acid alignments to nonprimate DUF1220 domains reveal that CON1 and CON2 are found in nonprimate mammals as well (data not shown). The other three clades, HLS1–3, typically fall between CON2 and CON3 in old world monkey, ape, and human *NBPF* genes but have been greatly expanded in copy number, specifically in the human lineage.

Comparison of this evolutionary profile of the *NBPF*-encoded DUF1220 domains with their gene organization indicates that, excluding four predicted pseudogenes, there are three types of functional *NBPF* genes in the human genome and that these exhibit widely different evolutionary histories from one another (Figure 2B and Table 2). First, the three genes that make up the group of nonprimate *NBPF* genes (*NBPF* 4, 6, and 7) exhibit a gene organization shared with nonprimate mammals, map exclusively to the human 1p-arm, and are composed entirely of conserved (CON1–3) DUF1220 domains. Second, the nine genes in the group of primate *NBPF* genes (*NBPF* 1, 3, 9, 11, 13, 24, 15, 16, and 23) show a type of gene organization that emerged only in the primate lineage and are located primarily in the 1q21.1–q21.2 region. These genes begin with N-terminal DUF1220 domains from the conserved CON1 and CON2 clades, followed by 1–5 HLS-type DUF1220 domains, and end with a C-terminal CON3 domain. Third, the six genes in the group of HLS *NBPF* genes (*NBPF* 8, 10, 12, 25, 14, and 20) are only found in the human lineage and map exclusively to the 1q21.1–q21.2 region. The most striking feature that distinguishes the HLS *NBPF* gene organization from the other two is that it encodes a hyperamplified number of HLS DUF1220 domains (7–48) in the middle of the gene between the CON2 and CON3 domains (Figure 2B and Table 2). Such intragenic domain hyperamplification is absent in all other primate genomes examined.

### HLS DUF1220 triplet

This phylogenetic profile also shows that 73% (199/272) of the human DUF1220 domains cluster to just the three HLS clades (HLS1–3) (Figure 2A). When the genomic positions of the DUF1220 domains that fall into these clades are compared with the predicted *NBPF* gene positions, 173 of the 199 HLS1–3 DUF1220 domains cluster to only 6 of the predicted 23 *NBPF* genes in the genome (Figure 3). Most strikingly, when the order of the HLS domains within each of these 6 HLS *NBPF* genes is examined, a tandemly repeated 4.7 kb triplet pattern, comprised of one member from each of the three HLS clades, emerges which we have named the *HLS DUF1220 triplet*. The triplet is made up of six exons and six introns, with 90% of the 4.7 kb being intronic sequence. Each triplet shows high similarity to one another, with members of the same HLS clade exhibiting 96–100% nucleotide sequence identify. Although HLS1 and HLS3 clades show the greatest interclade divergence among the HLS clades, they still maintain high sequence similarity to one another (85–90%).

It is noteworthy that although the *HLS DUF1220 triplet* is present in the chimp and gorilla genomes, indicating that the triplet arose prior to the split of human and gorilla, it did not undergo the pronounced expansion seen in the human lineage. In both of these genomes, the triplet is found only in the unexpanded primate *NBPF* form mentioned in the previous section. The largest predicted chimp and gorilla *NBPFs* have five of the HLS clade DUF1220s in a single complete gene, indicating that there has not been a triplet duplication within the chimp genome. This lack of triplet expansion is further supported by copy number estimates of DUF1220 domains based on sequence read depth (Sudmant *et al.* 2010), which estimates that copy number in chimp of the *HLS DUF1220 triplet*-containing gene *NBPF14* is a fifth of that in human.

**Figure 4:**
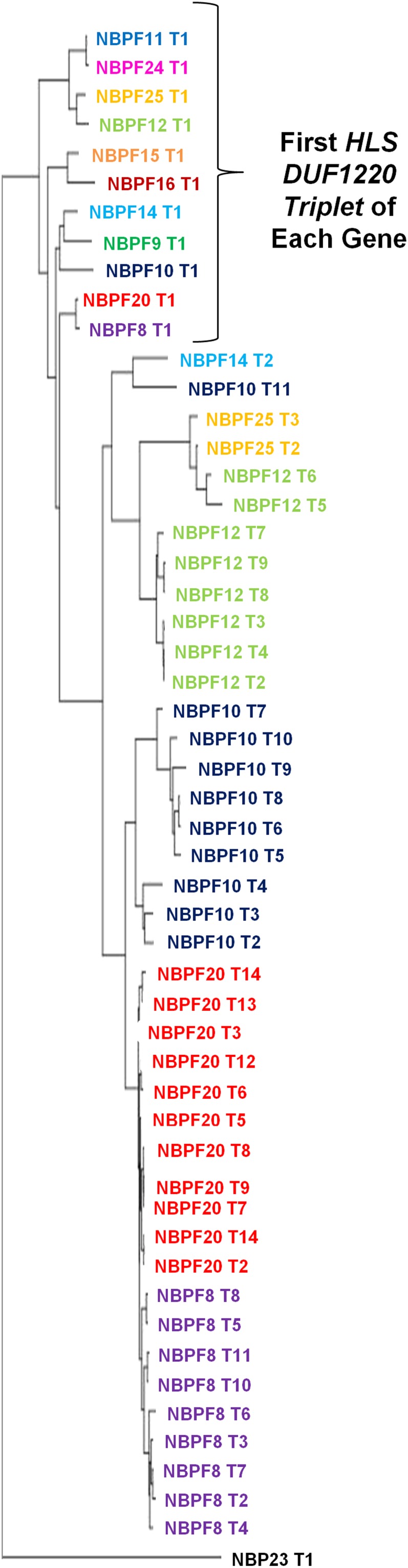
Evolutionary relationship of *HLS DUF1220 triplets.* Phylogenetic sequence-based alignments of the *HLS DUF1220 triplets* reveal that the first triplets in each NBPF gene are most similar to one another. Each successive triplet is then most similar to the triplets contained within each NBPF gene, indicating that the hyperamplification of the *HLS DUF1220 triplet* occurred as the result of intragenic domain amplification.

Interestingly, the DUF1220 domains within the *HLS DUF1220 triplet* clades are more closely related to other HLS triplet domains within the same gene than they are to the triplets in a different gene, indicating they arose predominantly via intragenic domain amplification rather than gene duplication (Figure 4). Remarkably, the intragenic hyperamplification of the *HLS DUF1220 triplet* added ∼149 DUF1220 copies to the human genome, whereas only ∼21 copies were added via gene duplication. Thus, in total, the *HLS DUF1220 triplet* domain hyperamplification accounts for 82% of the 182 HLS clade domain copies in the human 1q21 region and 88% (149/170) of the DUF1220 domains added to the human genome since our last common ancestor with the *Pan* genus (an average HLS gain of ∼28 DUF1220 copies every million years since our divergence with *Pan*).

## Discussion

The data presented here represent the most complete chronology of the evolutionary history of DUF1220 protein domains so far reported and include the identification of several key events related to the unusually rapid copy number amplification of these sequences. These include identification of a DUF1220 precursor dating as far back as ∼450 mya; the emergence of the first *NBPF* gene roughly ∼100–150 mya, which was likely due to the local duplication of a DUF1220-encoding segment of the *PDE4DIP* gene and its acquisition of the novel CM promoter; accelerated evolution of the DUF1220 domains in the primate lineage, including both gene and domain copy number increases; and the appearance of new DUF1220 clades.

The most striking finding, however, was the accelerated rise in copy number of the new DUF1220 domains in the human lineage due to intragenic domain hyperamplification involving the *HLS DUF1220 triplet*. To deduce a plausible explanation for this copy number increase in the human lineage, the *HLS DUF1220 triplet* sequences and their genomic locations were examined. Although the presence of transposable element (TE) insertions in intronic regions has been documented in other cases of domain amplification (Björklund *et al.* 2010), we found no evidence of insertions in any of the HLS triplets and hypothesize that the triplets may have something inherent to their sequence that makes them prone to amplification.

**Figure 5:**
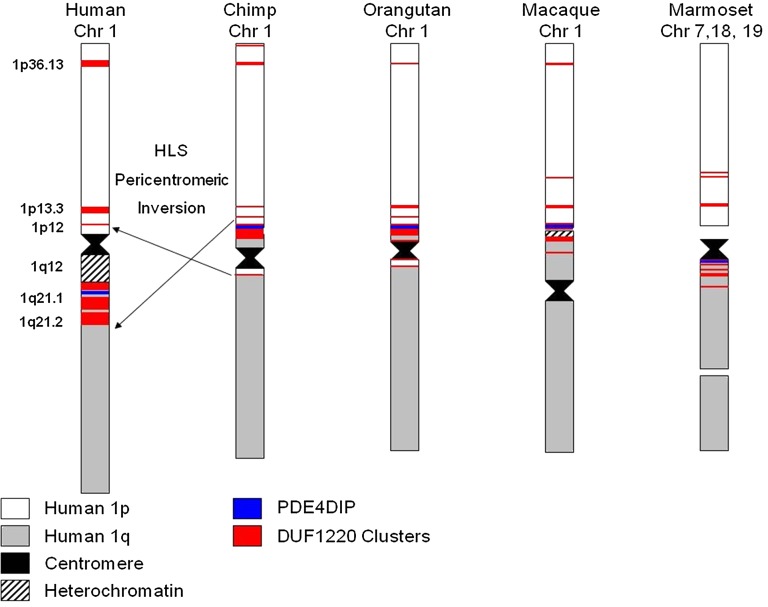
DUF1220 localization and cytogenetic architecture from five primate species. An ideogram depicting chromosome 1 of human, chimp, orangutan, macaque, and marmoset is shown, with arm color designations in relation to the human chromosomes 1p (white) and 1q (gray) gene order. Centromere position is indicated by a black block, and heterochromatin bands are indicated by black hash marks. The position of the *PDE4DIP* gene is indicated by a blue line, and clusters of DUF1220 copies are shown in red.

Mapping of the chromosomal locations of DUF1220 domains using the UCSC browser and data from Szamalek *et al.* (2006) (Figure 5) revealed that all copies of the *HLS DUF1220 triplet* lie in a unique human-specific genomic environment. Interestingly, although this analysis showed that all five primates tested have their largest cluster of DUF1220 domains at a homologous chromosomal position to human 1q21.1–q21.2, in human this entire region underwent an HLS pericentric inversion (1p11.2–q21.2). All *HLS DUF1220 triplets* map within this HLS inversion to a 7 Mb segment from 142.6 Mb to 149.7 Mb (Feb 2009 assembly, hg19). This interval along with the flanking 1q12 region was involved in the inversion, and both regions underwent dramatic human-specific genomic expansions (Figure 1B). 1q12, which has long been documented as a cytogenetically visible C-band, is a highly polymorphic, repeat-rich heterochromatic chromosomal band unique to humans that may have expanded after the inversion took place (Yunis and Prakash 1982). Both the 1q12 expansion and the 1p11.2–q21.2 inversion represent major alterations in the genome architecture of the pericentric region of human chromosome 1, and as such, may have played key roles in the rapid *HLS DUF1220 triplet* expansion.

A plausible explanation for these genomic changes focuses on the fact that when the inversion occurred early in the human lineage both inverted and noninverted chromosomes were present. As a result, in matings involving inverted/noninverted chromosomes, proper meiotic pairing and interchromosomal recombination of this region of chromosome 1 may have been temporarily impaired. In turn, this may have augmented allele fixation and led to several rounds of intrachromosomal nonallelic homologous recombination (NAHR), as has been observed for the Y chromosome (Rozen *et al.* 2003). The reduced interchromosomal allelic recombination may have favored enhanced rates of intrachromosomal NAHR that, combined with positive selection, may have triggered the fixation of chromosomes with intragenic DUF1220 gene and/or domain amplification(s). In this manner, the merging of these forces could have allowed DUF1220 domain copy number to hyperamplify, expanding from ∼102 DUF1220 copies in our last common ancestor with the *Pan* lineage to 272 in modern human.

Most importantly, these studies have laid the groundwork for future studies into DUF1220 function. Discovery of the six DUF1220 clades and their distinct evolutionary histories and gene positions indicates that they may have different functional roles within an NBPF protein. Future studies should focus on what roles the different clades play, especially the *NBPF* genes that contain the *HLS DUF1220 triplet*. In addition, selection for the amplification of the *HLS DUF1220 triplet* may indicate that the triplet itself confers a functional advantage, such as the super repeat seen in nebulin proteins, which has a higher binding affinity than a single nebulin repeat alone (Björklund *et al.* 2010).

In summary, the human-specific hyperamplification of the *HLS DUF1220 triplet* described here represents the primary mechanism responsible for the unprecedented DUF1220 copy number expansion in the human lineage. This rapid burst in copy number is likely to be highly adaptive within the human lineage, and it may have contributed to, and/or been facilitated by, the duplication-promoting genome architecture that characterizes the 1q21 region. As this genomic feature is only found in the human lineage, it is likely that only the human species bears the high recurrent disease burden that has been associated with the 1q21 region (Dumas and Sikela 2009).

See Table 3 for a glossary of terms used in this article.

**Table 3:** Glossary

DUF1220 Terminology	Description
CM promoter	Conserved mammalian promoter
CON1	Conserved clade; 1^st^ DUF1220 domain in NBPF genes
CON2	Conserved clade; 2^nd^ DUF1220 domain in NBPF genes
CON3	Conserved clade; last DUF1220 domain in NBPF genes
HLS1	Human Lineage-Specific expansion clade; 1^st^ member *HLS DUF1220 triplet*
HLS2	Human Lineage-Specific expansion clade; 2^nd^ member *HLS DUF1220 triplet*
HLS3	Human Lineage-Specific expansion clade; 3^rd^ member *HLS DUF1220 triplet*

## Supplementary Material

Supporting Information
