# Supplementary material for: Evolutionary History and Genome Organization of DUF1220 Protein Domains
Source: G3 (Bethesda). 2012 Sep 1;2(9):977–86. doi: 10.1534/g3.112.003061 (PMC3429928; doi:10.1534/g3.112.003061)
Supplement: Supporting Information [file supp_2_9_977__index.html]

Supporting Information 

# Evolutionary History and Genome Organization of DUF1220 Protein Domains

## Supporting Information for O'Bleness *et al.*, 2012

**Files in this Data Supplement:**

- Supporting Information - Figures S1 and S2 and Tables S1 and S2 (PDF, 382 KB)
- Figure S1 - Evolution of DUF1220 domain precursor in PDE4DIP (PDF, 244 KB)
- Figure S2 - Global alignment of a region 2000 bp upstream of predicted *NBPF* genes (PDF, 172 KB)
- Table S1 - Comparison of characteristics within the six primate DUF1220 clades (PDF, 50 KB)
- Table S2 - Correspondence of each superclade in the DUF1220 evolutionary phylogeny with the 10 Pfam DUF1220 seed domains (PDF, 53 KB)
