# Supplementary material for: Evolutionary History and Genome Organization of DUF1220 Protein Domains
Source: G3 (Bethesda). 2012 Sep 1;2(9):977–86. doi: 10.1534/g3.112.003061 (PMC3429928; doi:10.1534/g3.112.003061)
Supplement: Supporting Information [file supp_2.9.977_003061SI.pdf]

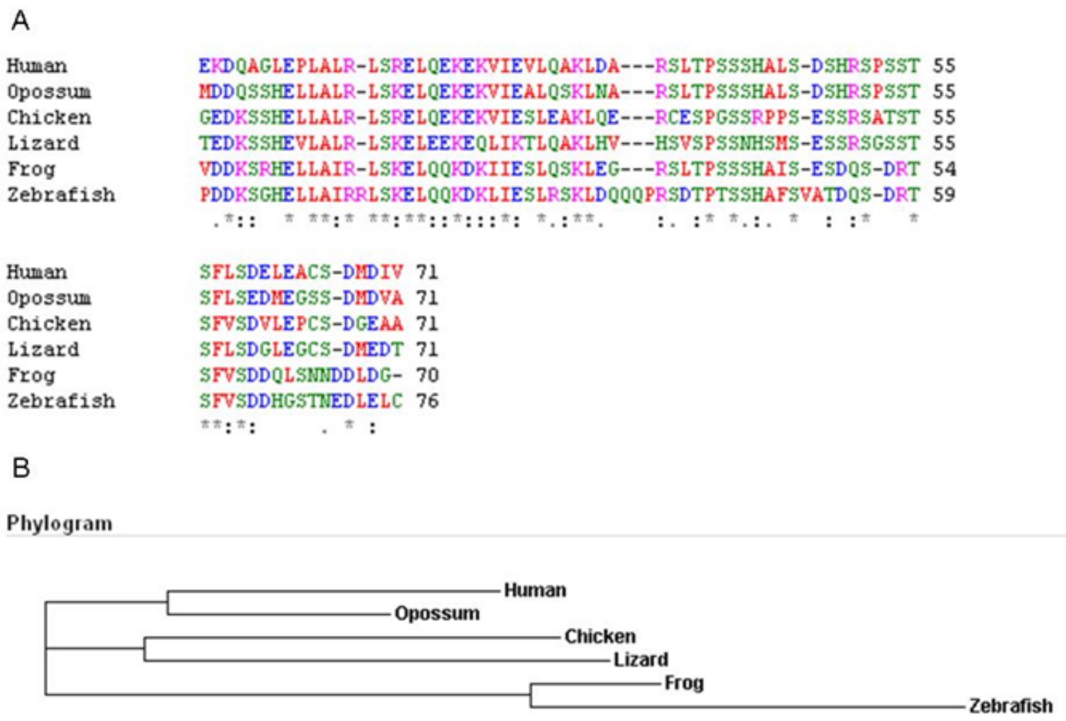

Figure S1 Evolution of DUF1220 domain precursor in PDE4DIP. (A) A sequence alignment performed with ClustalW of the DUF1220 protein domain homologous region in PDE4DIP in human, opossum, chicken, lizard, frog, and zebrafish. Only the region in human and opossum are conserved enough to meet criteria for being called a DUF1220 domain. However, there are several highly conserved amino acids and what appears to be a general progression in amino acid changes that show the eventual formation of the DUF1220 protein domain, as it is currently recognized. (B) Phylogenetic profile created by ClustalW of the sequence alignment shown in 2A. The phylogeny mirrors the known evolutionary relationships of these species, suggesting that the DUF1220 domain evolved over time from a precursor region in non-mammalian vertebrates prior to the appearance of the mammalian order.

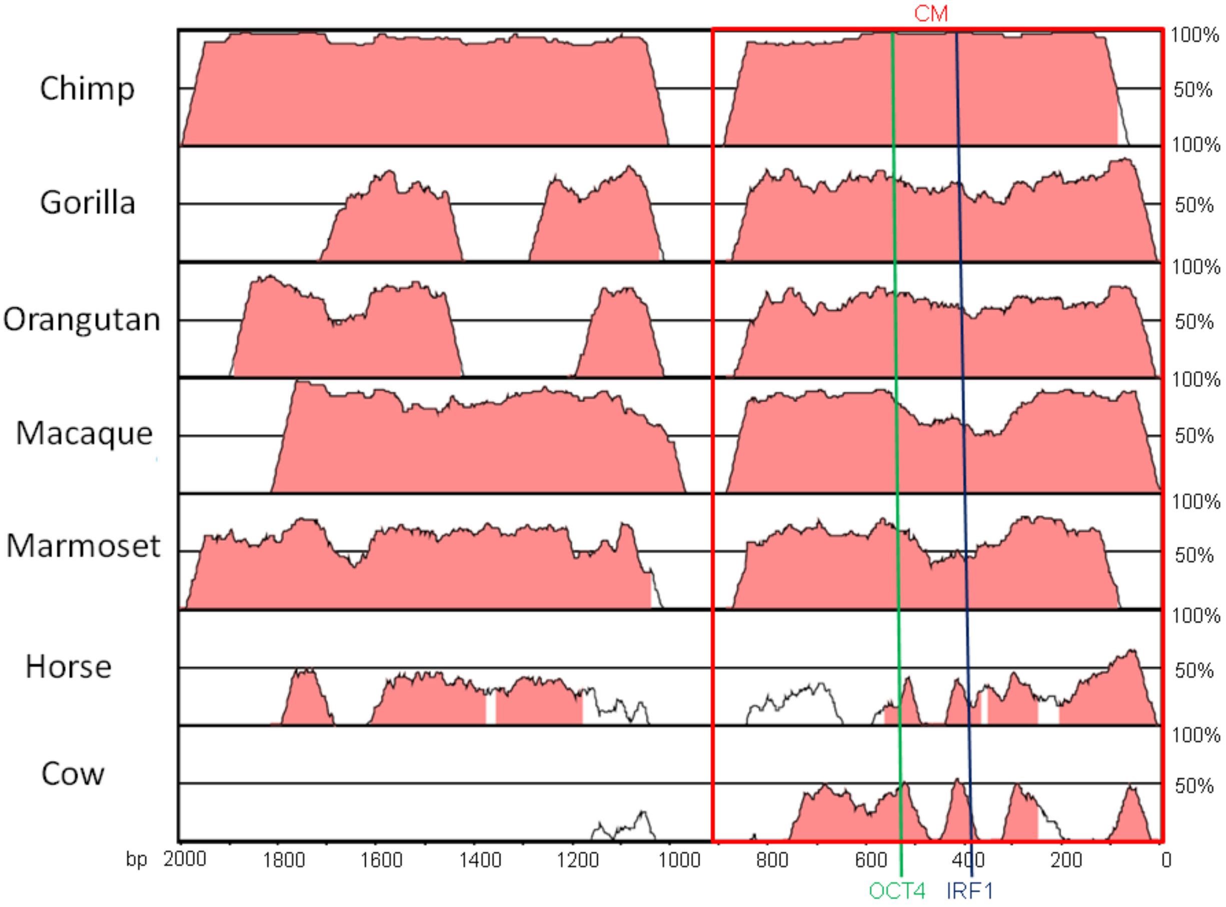

Figure S2 Global alignment of a region 2000 bp upstream of predicted *NBPF* genes. The upstream regions of predicted *NBPF* genes in 8 species were aligned to the 2000 bp upstream of human *NBPF*4 using the VISTA multiple alignment tool. The CM promoter is boxed in red with the two conserved transcription factor binding sites marked in green and dark blue.

Table S1   Comparison of characteristics within the six primate DUF1220 clades

Clades	Approximate Appearance (mya)	% Clade Human	Copies per Gene	Copies in Human Genome	Placement of DUF1220 within NBPF Gene	Primary Mech. Clade Emergence
CON1	>90	44	1-3	36	N-terminal	Gene Dup
CON2	>90	32	1	20	Next to CON1	Gene Dup
CON3	>40	27	0-1	15	C-terminal	Gene Dup
HLS1	25-40	72	0-14	61	Between CON2 & CON3	Domain Dup
HLS2	25-40	66	0-17	71	Between CON2 & CON3	Domain Dup
HLS3	25-40	62	0-17	67	Between CON2 & CON3	Domain Dup

Table S2 Correspondence of each superclade in the DUF1220 evolutionary phylogeny with the 10 Pfam DUF1220 seed domains.

Pfam Seed Domain	Superclade
Q8IX62 111-177	HLS1
Q8IX62 186-252	HLS2
Q8ND86 36-102	HLS2
Q8IX62 17-83	HLS3
Q9C0H0 138-201	CON1
Q9H094 236-298	CON1
Q8IX77 116-178	CON1
Q8IX71 95-158	CON2
095877 28-94	CON3
Q8ND86 186-252	CON3
