# Supplementary material for: Evolutionary History and Genome Organization of DUF1220 Protein Domains
Source: G3 (Bethesda). 2012 Sep 1;2(9):977–86. doi: 10.1534/g3.112.003061 (PMC3429928; doi:10.1534/g3.112.003061)
Supplement: Supporting Information [file supp_2.9.977_FigureS1.pdf]

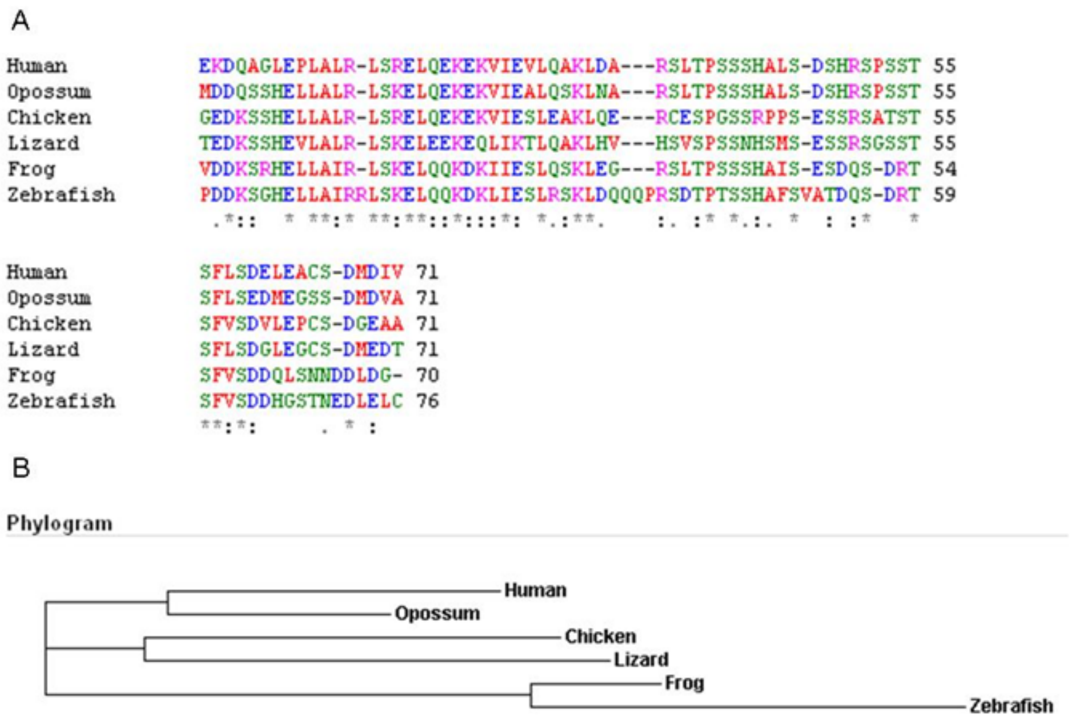

Figure S1 Evolution of DUF1220 domain precursor in PDE4DIP. (A) A sequence alignment performed with ClustalW of the DUF1220 protein domain homologous region in PDE4DIP in human, opossum, chicken, lizard, frog, and zebrafish. Only the region in human and opossum are conserved enough to meet criteria for being called a DUF1220 domain. However, there are several highly conserved amino acids and what appears to be a general progression in amino acid changes that show the eventual formation of the DUF1220 protein domain, as it is currently recognized. (B) Phylogenetic profile created by ClustalW of the sequence alignment shown in 2A. The phylogeny mirrors the known evolutionary relationships of these species, suggesting that the DUF1220 domain evolved over time from a precursor region in non-mammalian vertebrates prior to the appearance of the mammalian order.
