# Supplementary material for: Evolutionary History and Genome Organization of DUF1220 Protein Domains
Source: G3 (Bethesda). 2012 Sep 1;2(9):977–86. doi: 10.1534/g3.112.003061 (PMC3429928; doi:10.1534/g3.112.003061)
Supplement: Supporting Information [file supp_2.9.977_FigureS2.pdf]

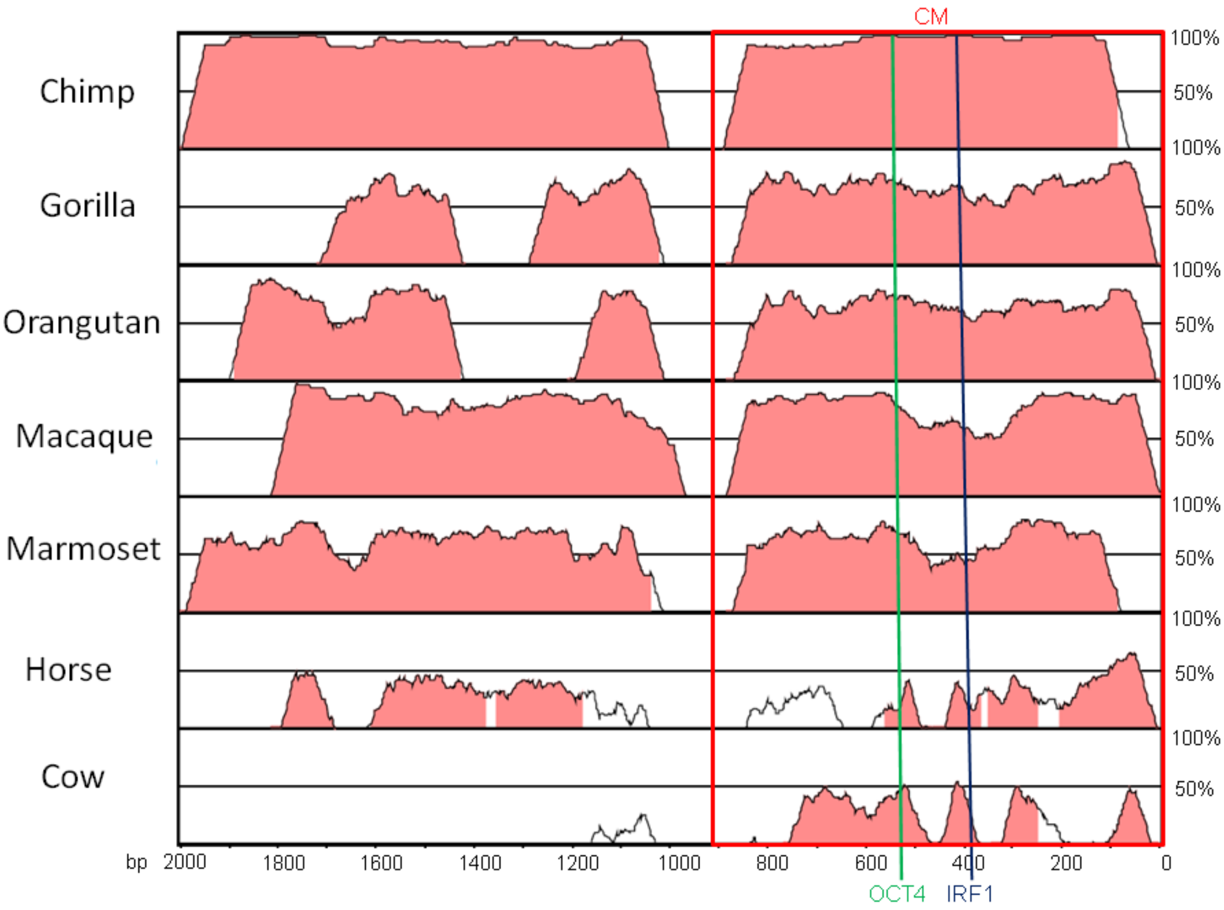

Figure S2 Global alignment of a region 2000 bp upstream of predicted *NBPF* genes. The upstream regions of predicted *NBPF* genes in 8 species were aligned to the 2000 bp upstream of human *NBPF*4 using the VISTA multiple alignment tool. The CM promoter is boxed in red with the two conserved transcription factor binding sites marked in green and dark blue.
