# Supplementary material for: Evolutionary History and Genome Organization of DUF1220 Protein Domains
Source: G3 (Bethesda). 2012 Sep 1;2(9):977–86. doi: 10.1534/g3.112.003061 (PMC3429928; doi:10.1534/g3.112.003061)
Supplement: Supporting Information [file supp_2.9.977_TableS1.pdf]

**Table S1   Comparison of characteristics within the six primate DUF1220 clades**

| Clades | Approximate Appearance (mya) | % Clade Human | Copies per Gene | Copies in Human Genome | Placement of DUF1220 within NBPF Gene | Primary Mech. Clade Emergence |
|---|---|---|---|---|---|---|
| CON1 | >90 | 44 | 1-3 | 36 | N-terminal | Gene Dup |
| CON2 | >90 | 32 | 1 | 20 | Next to CON1 | Gene Dup |
| CON3 | >40 | 27 | 0-1 | 15 | C-terminal | Gene Dup |
| HLS1 | 25-40 | 72 | 0-14 | 61 | Between CON2 & CON3 | Domain Dup |
| HLS2 | 25-40 | 66 | 0-17 | 71 | Between CON2 & CON3 | Domain Dup |
| HLS3 | 25-40 | 62 | 0-17 | 67 | Between CON2 & CON3 | Domain Dup |
