# Supplementary material for: Evolutionary History and Genome Organization of DUF1220 Protein Domains
Source: G3 (Bethesda). 2012 Sep 1;2(9):977–86. doi: 10.1534/g3.112.003061 (PMC3429928; doi:10.1534/g3.112.003061)
Supplement: Supporting Information [file supp_2.9.977_TableS2.pdf]

Table S2 Correspondence of each superclade in the DUF1220 evolutionary phylogeny with the 10 Pfam DUF1220 seed domains.

Pfam Seed Domain	Superclade
Q8IX62 111-177	HLS1
Q8IX62 186-252	HLS2
Q8ND86 36-102	HLS2
Q8IX62 17-83	HLS3
Q9C0H0 138-201	CON1
Q9H094 236-298	CON1
Q8IX77 116-178	CON1
Q8IX71 95-158	CON2
095877 28-94	CON3
Q8ND86 186-252	CON3
